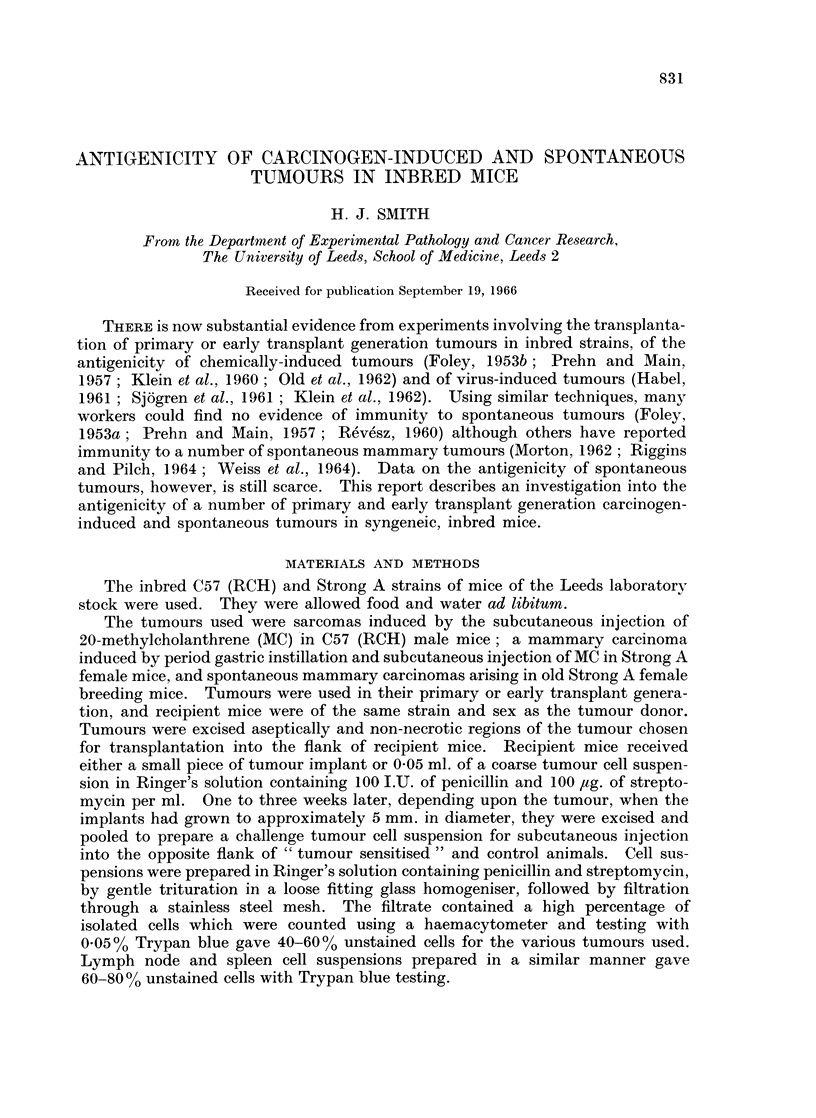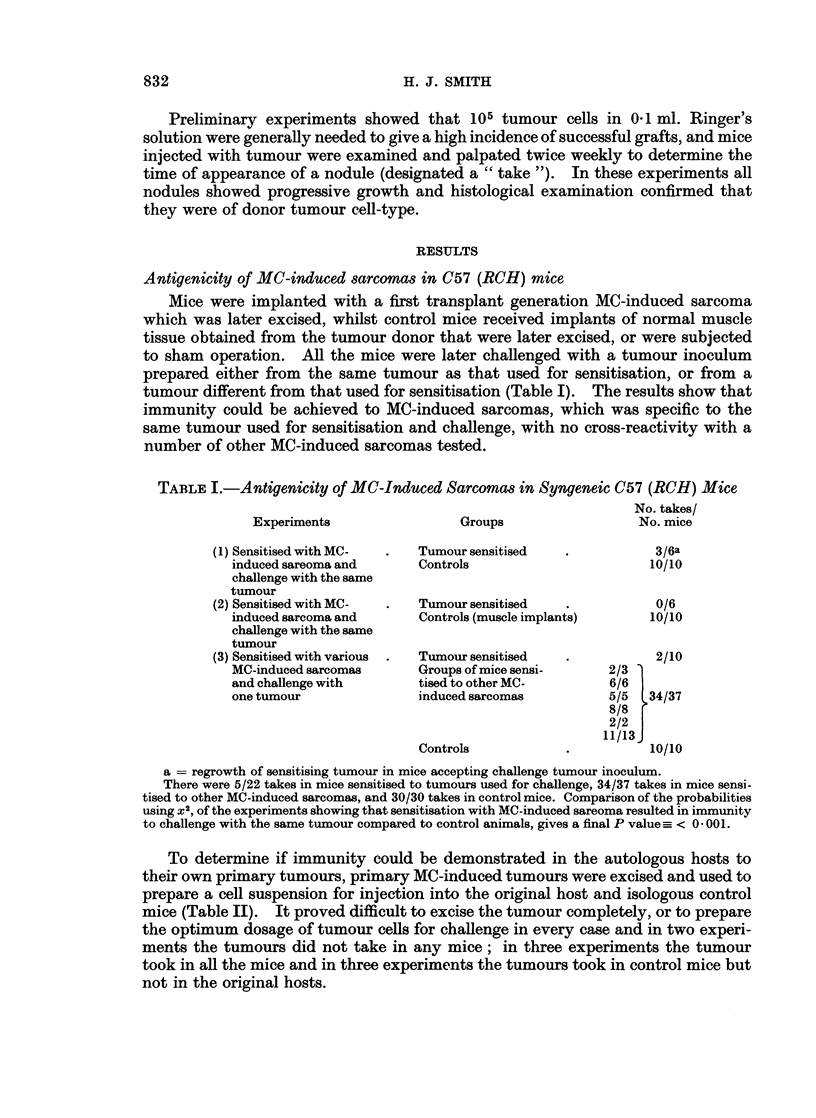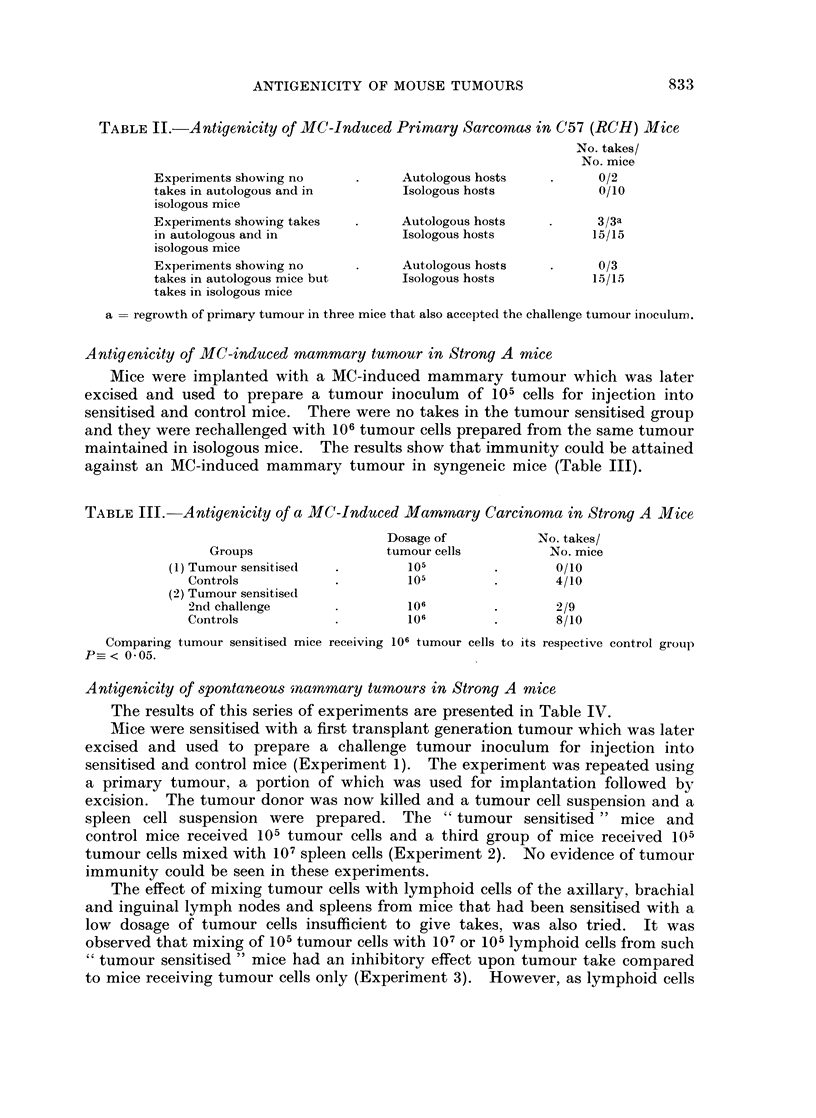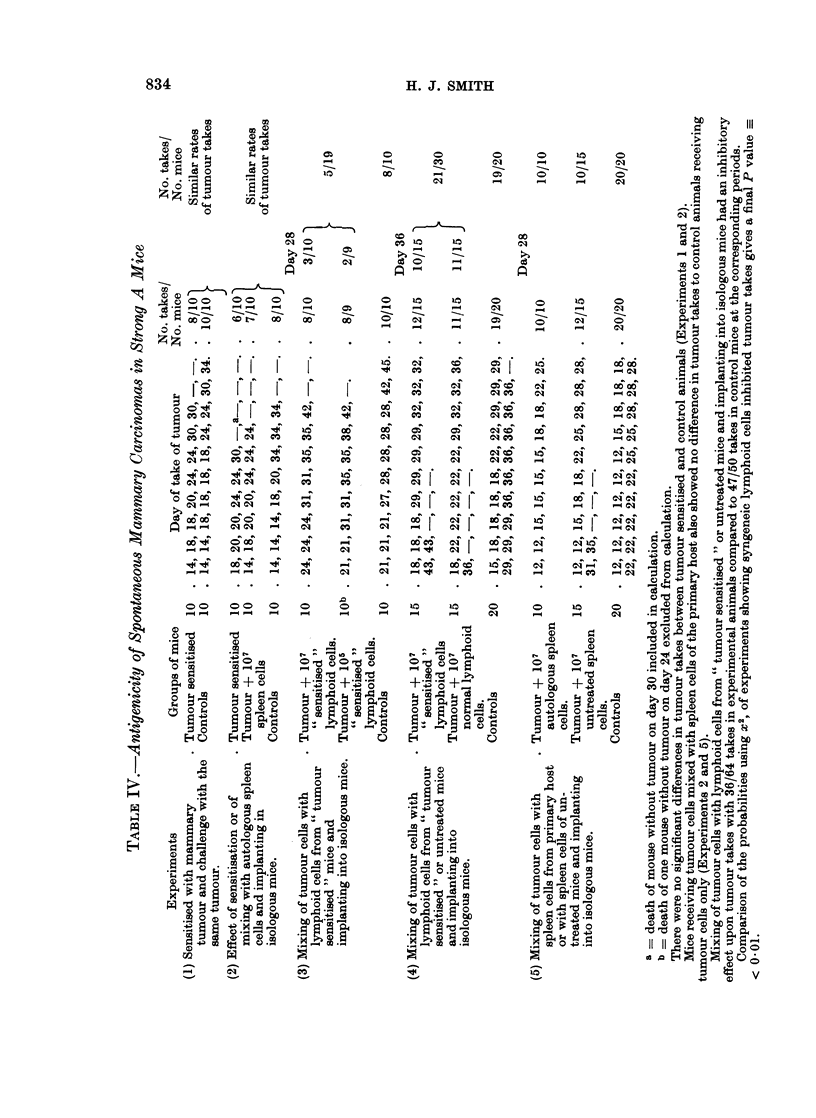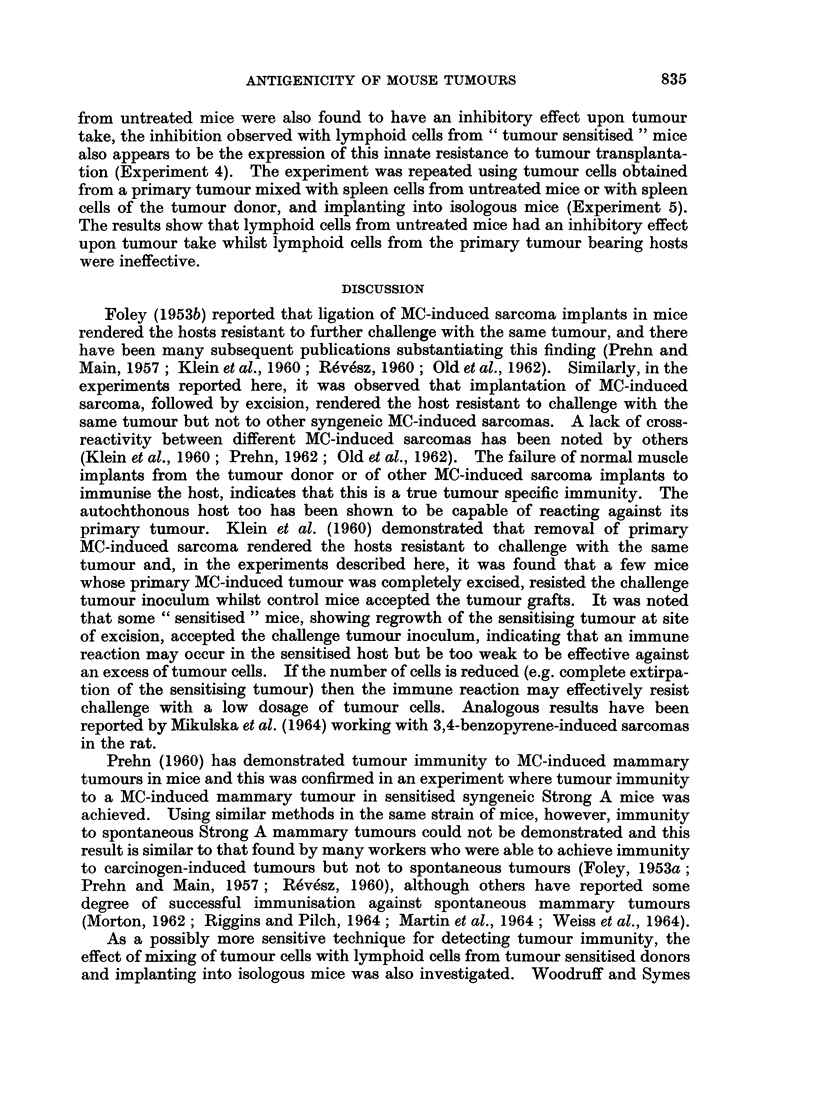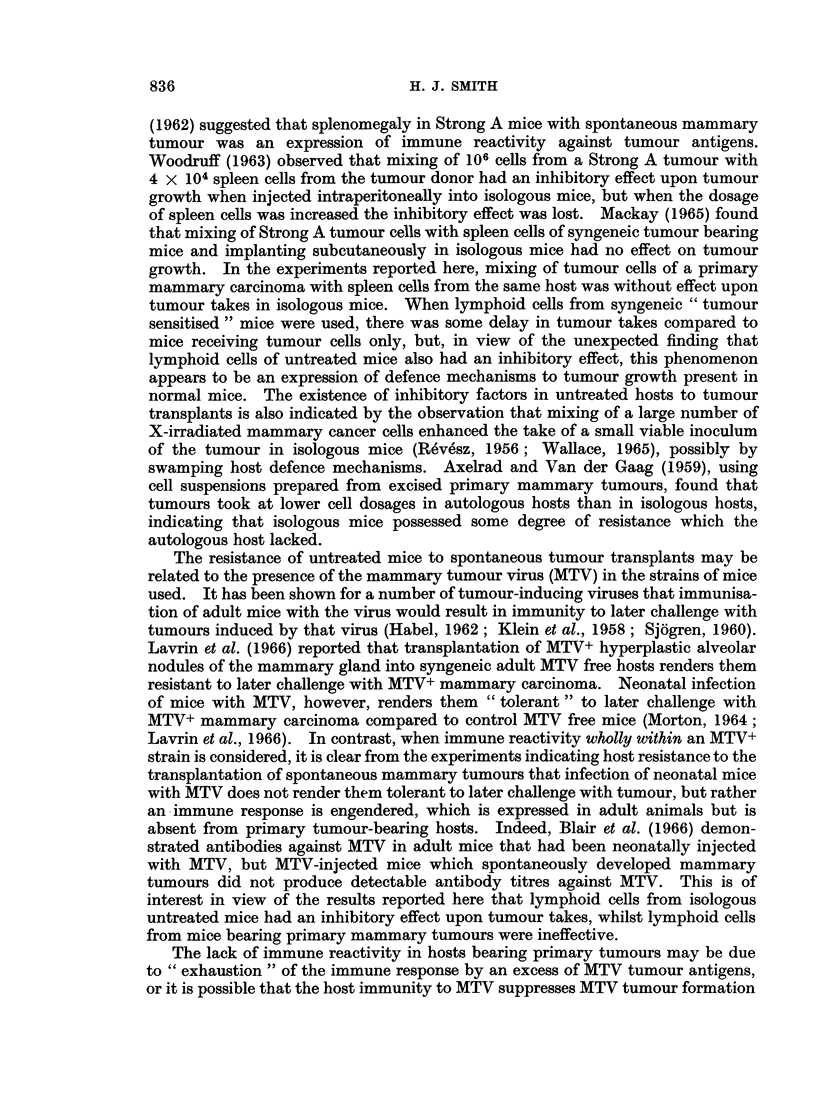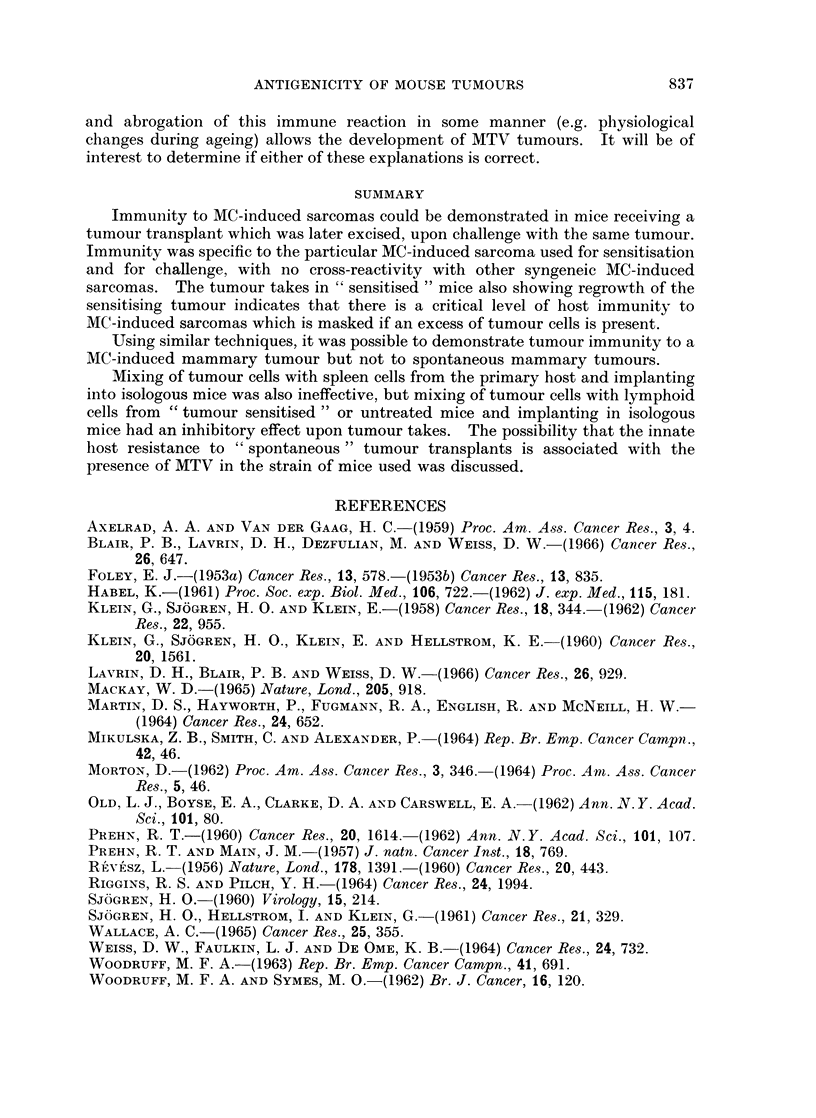# Antigenicity of carcinogen-induced and spontaneous tumours in inbred mice.

**DOI:** 10.1038/bjc.1966.95

**Published:** 1966-12

**Authors:** H. J. Smith


### ANTIGENICITY OF CARCINOGEN-INDUCED AND SPONTANEOUS TUMOURS IN INBRED MICE

H. J. SMITH

*From the Department of Experimental Pathology and Cancer Research, The University of Leeds, School of Medicine, Leeds 2*

Received for publication September 19, 1966

THERE is now substantial evidence from experiments involving the transplantation of primary or early transplant generation tumours in inbred strains, of the antigenicity of chemically-induced tumours (Foley, 1953*b* ; Prehn and Main, 1957 ; Klein *et al.*, 1960 ; Old *et al.*, 1962) and of virus-induced tumours (Habel, 1961 ; Sjögren *et al.*, 1961 ; Klein *et al.*, 1962). Using similar techniques, many workers could find no evidence of immunity to spontaneous tumours (Foley, 1953*a* ; Prehn and Main, 1957 ; Révész, 1960) although others have reported immunity to a number of spontaneous mammary tumours (Morton, 1962 ; Riggins and Pilch, 1964 ; Weiss *et al.*, 1964). Data on the antigenicity of spontaneous tumours, however, is still scarce. This report describes an investigation into the antigenicity of a number of primary and early transplant generation carcinogen-induced and spontaneous tumours in syngeneic, inbred mice.

### MATERIALS AND METHODS

The inbred C57 (RCH) and Strong A strains of mice of the Leeds laboratory stock were used. They were allowed food and water *ad libitum*.

The tumours used were sarcomas induced by the subcutaneous injection of 20-methylcholanthrene (MC) in C57 (RCH) male mice ; a mammary carcinoma induced by period gastric instillation and subcutaneous injection of MC in Strong A female mice, and spontaneous mammary carcinomas arising in old Strong A female breeding mice. Tumours were used in their primary or early transplant generation, and recipient mice were of the same strain and sex as the tumour donor. Tumours were excised aseptically and non-necrotic regions of the tumour chosen for transplantation into the flank of recipient mice. Recipient mice received either a small piece of tumour implant or 0·05 ml. of a coarse tumour cell suspension in Ringer's solution containing 100 I.U. of penicillin and 100 $\mu$g. of streptomycin per ml. One to three weeks later, depending upon the tumour, when the implants had grown to approximately 5 mm. in diameter, they were excised and pooled to prepare a challenge tumour cell suspension for subcutaneous injection into the opposite flank of " tumour sensitised " and control animals. Cell suspensions were prepared in Ringer's solution containing penicillin and streptomycin, by gentle trituration in a loose fitting glass homogeniser, followed by filtration through a stainless steel mesh. The filtrate contained a high percentage of isolated cells which were counted using a haemacytometer and testing with 0·05% Trypan blue gave 40–60% unstained cells for the various tumours used. Lymph node and spleen cell suspensions prepared in a similar manner gave 60–80% unstained cells with Trypan blue testing.

Preliminary experiments showed that $10^5$ tumour cells in 0·1 ml. Ringer's solution were generally needed to give a high incidence of successful grafts, and mice injected with tumour were examined and palpated twice weekly to determine the time of appearance of a nodule (designated a " take "). In these experiments all nodules showed progressive growth and histological examination confirmed that they were of donor tumour cell-type.

### RESULTS

### *Antigenicity of MC-induced sarcomas in C57 (RCH) mice*

Mice were implanted with a first transplant generation MC-induced sarcoma which was later excised, whilst control mice received implants of normal muscle tissue obtained from the tumour donor that were later excised, or were subjected to sham operation. All the mice were later challenged with a tumour inoculum prepared either from the same tumour as that used for sensitisation, or from a tumour different from that used for sensitisation (Table I). The results show that immunity could be achieved to MC-induced sarcomas, which was specific to the same tumour used for sensitisation and challenge, with no cross-reactivity with a number of other MC-induced sarcomas tested.

TABLE I.—*Antigenicity of MC-Induced Sarcomas in Syngeneic C57 (RCH) Mice*

| Experiments | Groups | | No. takes/ No. mice |
|---|---|---|---|
| (1) Sensitised with MC-induced sareoma and challenge with the same tumour | Tumour sensitised | | 3/6[a] |
| | Controls | | 10/10 |
| (2) Sensitised with MC-induced sarcoma and challenge with the same tumour | Tumour sensitised | | 0/6 |
| | Controls (muscle implants) | | 10/10 |
| (3) Sensitised with various MC-induced sarcomas and challenge with one tumour | Tumour sensitised | | 2/10 |
| | Groups of mice sensitised to other MC-induced sarcomas | 2/3, 6/6, 5/5, 8/8, 2/2, 11/13 | 34/37 |
| | Controls | | 10/10 |

a = regrowth of sensitising tumour in mice accepting challenge tumour inoculum.

There were 5/22 takes in mice sensitised to tumours used for challenge, 34/37 takes in mice sensitised to other MC-induced sarcomas, and 30/30 takes in control mice. Comparison of the probabilities using $x^2$, of the experiments showing that sensitisation with MC-induced sareoma resulted in immunity to challenge with the same tumour compared to control animals, gives a final $P$ value $\equiv < 0 \cdot 001$.

To determine if immunity could be demonstrated in the autologous hosts to their own primary tumours, primary MC-induced tumours were excised and used to prepare a cell suspension for injection into the original host and isologous control mice (Table II). It proved difficult to excise the tumour completely, or to prepare the optimum dosage of tumour cells for challenge in every case and in two experiments the tumours did not take in any mice ; in three experiments the tumour took in all the mice and in three experiments the tumours took in control mice but not in the original hosts.

TABLE II.—*Antigenicity of MC-Induced Primary Sarcomas in C57 (RCH) Mice*

| | | No. takes/ No. mice |
|---|---|---|
| Experiments showing no takes in autologous and in isologous mice | Autologous hosts | 0/2 |
| | Isologous hosts | 0/10 |
| Experiments showing takes in autologous and in isologous mice | Autologous hosts | 3/3[a] |
| | Isologous hosts | 15/15 |
| Experiments showing no takes in autologous mice but takes in isologous mice | Autologous hosts | 0/3 |
| | Isologous hosts | 15/15 |

a = regrowth of primary tumour in three mice that also accepted the challenge tumour inoculum.

*Antigenicity of MC-induced mammary tumour in Strong A mice*

Mice were implanted with a MC-induced mammary tumour which was later excised and used to prepare a tumour inoculum of $10^5$ cells for injection into sensitised and control mice. There were no takes in the tumour sensitised group and they were rechallenged with $10^6$ tumour cells prepared from the same tumour maintained in isologous mice. The results show that immunity could be attained against an MC-induced mammary tumour in syngeneic mice (Table III).

TABLE III.—*Antigenicity of a MC-Induced Mammary Carcinoma in Strong A Mice*

| Groups | Dosage of tumour cells | No. takes/ No. mice |
|---|---|---|
| (1) Tumour sensitised | $10^5$ | 0/10 |
| Controls | $10^5$ | 4/10 |
| (2) Tumour sensitised | | |
| 2nd challenge | $10^6$ | 2/9 |
| Controls | $10^6$ | 8/10 |

Comparing tumour sensitised mice receiving $10^6$ tumour cells to its respective control group $P \equiv < 0 \cdot 05$.

*Antigenicity of spontaneous mammary tumours in Strong A mice*

The results of this series of experiments are presented in Table IV.

Mice were sensitised with a first transplant generation tumour which was later excised and used to prepare a challenge tumour inoculum for injection into sensitised and control mice (Experiment 1). The experiment was repeated using a primary tumour, a portion of which was used for implantation followed by excision. The tumour donor was now killed and a tumour cell suspension and a spleen cell suspension were prepared. The " tumour sensitised " mice and control mice received $10^5$ tumour cells and a third group of mice received $10^5$ tumour cells mixed with $10^7$ spleen cells (Experiment 2). No evidence of tumour immunity could be seen in these experiments.

The effect of mixing tumour cells with lymphoid cells of the axillary, brachial and inguinal lymph nodes and spleens from mice that had been sensitised with a low dosage of tumour cells insufficient to give takes, was also tried. It was observed that mixing of $10^5$ tumour cells with $10^7$ or $10^5$ lymphoid cells from such " tumour sensitised " mice had an inhibitory effect upon tumour take compared to mice receiving tumour cells only (Experiment 3). However, as lymphoid cells from untreated mice were also found to have an inhibitory effect upon tumour take, the inhibition observed with lymphoid cells from " tumour sensitised " mice also appears to be the expression of this innate resistance to tumour transplantation (Experiment 4). The experiment was repeated using tumour cells obtained from a primary tumour mixed with spleen cells from untreated mice or with spleen cells of the tumour donor, and implanting into isologous mice (Experiment 5). The results show that lymphoid cells from untreated mice had an inhibitory effect upon tumour take whilst lymphoid cells from the primary tumour bearing hosts were ineffective.

### DISCUSSION

Foley (1953*b*) reported that ligation of MC-induced sarcoma implants in mice rendered the hosts resistant to further challenge with the same tumour, and there have been many subsequent publications substantiating this finding (Prehn and Main, 1957 ; Klein *et al.*, 1960 ; Révész, 1960 ; Old *et al.*, 1962). Similarly, in the experiments reported here, it was observed that implantation of MC-induced sarcoma, followed by excision, rendered the host resistant to challenge with the same tumour but not to other syngeneic MC-induced sarcomas. A lack of cross-reactivity between different MC-induced sarcomas has been noted by others (Klein *et al.*, 1960 ; Prehn, 1962 ; Old *et al.*, 1962). The failure of normal muscle implants from the tumour donor or of other MC-induced sarcoma implants to immunise the host, indicates that this is a true tumour specific immunity. The autochthonous host too has been shown to be capable of reacting against its primary tumour. Klein *et al.* (1960) demonstrated that removal of primary MC-induced sarcoma rendered the hosts resistant to challenge with the same tumour and, in the experiments described here, it was found that a few mice whose primary MC-induced tumour was completely excised, resisted the challenge tumour inoculum whilst control mice accepted the tumour grafts. It was noted that some " sensitised " mice, showing regrowth of the sensitising tumour at site of excision, accepted the challenge tumour inoculum, indicating that an immune reaction may occur in the sensitised host but be too weak to be effective against an excess of tumour cells. If the number of cells is reduced (e.g. complete extirpation of the sensitising tumour) then the immune reaction may effectively resist challenge with a low dosage of tumour cells. Analogous results have been reported by Mikulska *et al.* (1964) working with 3,4-benzopyrene-induced sarcomas in the rat.

Prehn (1960) has demonstrated tumour immunity to MC-induced mammary tumours in mice and this was confirmed in an experiment where tumour immunity to a MC-induced mammary tumour in sensitised syngeneic Strong A mice was achieved. Using similar methods in the same strain of mice, however, immunity to spontaneous Strong A mammary tumours could not be demonstrated and this result is similar to that found by many workers who were able to achieve immunity to carcinogen-induced tumours but not to spontaneous tumours (Foley, 1953*a* ; Prehn and Main, 1957 ; Révész, 1960), although others have reported some degree of successful immunisation against spontaneous mammary tumours (Morton, 1962 ; Riggins and Pilch, 1964 ; Martin *et al.*, 1964 ; Weiss *et al.*, 1964).

As a possibly more sensitive technique for detecting tumour immunity, the effect of mixing of tumour cells with lymphoid cells from tumour sensitised donors and implanting into isologous mice was also investigated. Woodruff and Symes

(1962) suggested that splenomegaly in Strong A mice with spontaneous mammary tumour was an expression of immune reactivity against tumour antigens. Woodruff (1963) observed that mixing of $10^6$ cells from a Strong A tumour with $4 \times 10^4$ spleen cells from the tumour donor had an inhibitory effect upon tumour growth when injected intraperitoneally into isologous mice, but when the dosage of spleen cells was increased the inhibitory effect was lost. Mackay (1965) found that mixing of Strong A tumour cells with spleen cells of syngeneic tumour bearing mice and implanting subcutaneously in isologous mice had no effect on tumour growth. In the experiments reported here, mixing of tumour cells of a primary mammary carcinoma with spleen cells from the same host was without effect upon tumour takes in isologous mice. When lymphoid cells from syngeneic " tumour sensitised " mice were used, there was some delay in tumour takes compared to mice receiving tumour cells only, but, in view of the unexpected finding that lymphoid cells of untreated mice also had an inhibitory effect, this phenomenon appears to be an expression of defence mechanisms to tumour growth present in normal mice. The existence of inhibitory factors in untreated hosts to tumour transplants is also indicated by the observation that mixing of a large number of X-irradiated mammary cancer cells enhanced the take of a small viable inoculum of the tumour in isologous mice (Révész, 1956; Wallace, 1965), possibly by swamping host defence mechanisms. Axelrad and Van der Gaag (1959), using cell suspensions prepared from excised primary mammary tumours, found that tumours took at lower cell dosages in autologous hosts than in isologous hosts, indicating that isologous mice possessed some degree of resistance which the autologous host lacked.

The resistance of untreated mice to spontaneous tumour transplants may be related to the presence of the mammary tumour virus (MTV) in the strains of mice used. It has been shown for a number of tumour-inducing viruses that immunisation of adult mice with the virus would result in immunity to later challenge with tumours induced by that virus (Habel, 1962; Klein *et al.*, 1958; Sjögren, 1960). Lavrin *et al.* (1966) reported that transplantation of $MTV^+$ hyperplastic alveolar nodules of the mammary gland into syngeneic adult MTV free hosts renders them resistant to later challenge with $MTV^+$ mammary carcinoma. Neonatal infection of mice with MTV, however, renders them " tolerant " to later challenge with $MTV^+$ mammary carcinoma compared to control MTV free mice (Morton, 1964; Lavrin *et al.*, 1966). In contrast, when immune reactivity *wholly within* an $MTV^+$ strain is considered, it is clear from the experiments indicating host resistance to the transplantation of spontaneous mammary tumours that infection of neonatal mice with MTV does not render them tolerant to later challenge with tumour, but rather an immune response is engendered, which is expressed in adult animals but is absent from primary tumour-bearing hosts. Indeed, Blair *et al.* (1966) demonstrated antibodies against MTV in adult mice that had been neonatally injected with MTV, but MTV-injected mice which spontaneously developed mammary tumours did not produce detectable antibody titres against MTV. This is of interest in view of the results reported here that lymphoid cells from isologous untreated mice had an inhibitory effect upon tumour takes, whilst lymphoid cells from mice bearing primary mammary tumours were ineffective.

The lack of immune reactivity in hosts bearing primary tumours may be due to " exhaustion " of the immune response by an excess of MTV tumour antigens, or it is possible that the host immunity to MTV suppresses MTV tumour formation and abrogation of this immune reaction in some manner (e.g. physiological changes during ageing) allows the development of MTV tumours. It will be of interest to determine if either of these explanations is correct.

### SUMMARY

Immunity to MC-induced sarcomas could be demonstrated in mice receiving a tumour transplant which was later excised, upon challenge with the same tumour. Immunity was specific to the particular MC-induced sarcoma used for sensitisation and for challenge, with no cross-reactivity with other syngeneic MC-induced sarcomas. The tumour takes in " sensitised " mice also showing regrowth of the sensitising tumour indicates that there is a critical level of host immunity to MC-induced sarcomas which is masked if an excess of tumour cells is present.

Using similar techniques, it was possible to demonstrate tumour immunity to a MC-induced mammary tumour but not to spontaneous mammary tumours.

Mixing of tumour cells with spleen cells from the primary host and implanting into isologous mice was also ineffective, but mixing of tumour cells with lymphoid cells from " tumour sensitised " or untreated mice and implanting in isologous mice had an inhibitory effect upon tumour takes. The possibility that the innate host resistance to " spontaneous " tumour transplants is associated with the presence of MTV in the strain of mice used was discussed.

### REFERENCES

Axelrad, A. A. and Van der Gaag, H. C.—(1959) *Proc. Am. Ass. Cancer Res.*, **3**, 4.
Blair, P. B., Lavrin, D. H., Dezfulian, M. and Weiss, D. W.—(1966) *Cancer Res.*, **26**, 647.
Foley, E. J.—(1953*a*) *Cancer Res.*, **13**, 578.—(1953*b*) *Cancer Res.*, **13**, 835.
Habel, K.—(1961) *Proc. Soc. exp. Biol. Med.*, **106**, 722.—(1962) *J. exp. Med.*, **115**, 181.
Klein, G., Sjögren, H. O. and Klein, E.—(1958) *Cancer Res.*, **18**, 344.—(1962) *Cancer Res.*, **22**, 955.
Klein, G., Sjögren, H. O., Klein, E. and Hellstrom, K. E.—(1960) *Cancer Res.*, **20**, 1561.
Lavrin, D. H., Blair, P. B. and Weiss, D. W.—(1966) *Cancer Res.*, **26**, 929.
Mackay, W. D.—(1965) *Nature, Lond.*, **205**, 918.
Martin, D. S., Hayworth, P., Fugmann, R. A., English, R. and McNeill, H. W.—(1964) *Cancer Res.*, **24**, 652.
Mikulska, Z. B., Smith, C. and Alexander, P.—(1964) *Rep. Br. Emp. Cancer Campn.*, **42**, 46.
Morton, D.—(1962) *Proc. Am. Ass. Cancer Res.*, **3**, 346.—(1964) *Proc. Am. Ass. Cancer Res.*, **5**, 46.
Old, L. J., Boyse, E. A., Clarke, D. A. and Carswell, E. A.—(1962) *Ann. N.Y. Acad. Sci.*, **101**, 80.
Prehn, R. T.—(1960) *Cancer Res.*, **20**, 1614.—(1962) *Ann. N.Y. Acad. Sci.*, **101**, 107.
Prehn, R. T. and Main, J. M.—(1957) *J. natn. Cancer Inst.*, **18**, 769.
Révész, L.—(1956) *Nature, Lond.*, **178**, 1391.—(1960) *Cancer Res.*, **20**, 443.
Riggins, R. S. and Pilch, Y. H.—(1964) *Cancer Res.*, **24**, 1994.
Sjögren, H. O.—(1960) *Virology*, **15**, 214.
Sjögren, H. O., Hellstrom, I. and Klein, G.—(1961) *Cancer Res.*, **21**, 329.
Wallace, A. C.—(1965) *Cancer Res.*, **25**, 355.
Weiss, D. W., Faulkin, L. J. and De Ome, K. B.—(1964) *Cancer Res.*, **24**, 732.
Woodruff, M. F. A.—(1963) *Rep. Br. Emp. Cancer Campn.*, **41**, 691.
Woodruff, M. F. A. and Symes, M. O.—(1962) *Br. J. Cancer*, **16**, 120.